# Cell-of-Origin Targeted Drug Repurposing for Triple-Negative and Inflammatory Breast Carcinoma with HDAC and HSP90 Inhibitors Combined with Niclosamide

**DOI:** 10.3390/cancers15020332

**Published:** 2023-01-04

**Authors:** Udayan Bhattacharya, Mohammad Kamran, Maroua Manai, Massimo Cristofanilli, Tan A. Ince

**Affiliations:** 1 Weill Cornell Medicine, Department of Pathology and Laboratory Medicine, New York, NY 10065, USA; 2 Weill Cornell Medicine, Division of Hematology-Oncology, New York, NY 10065, USA; 3 New York Presbyterian Brooklyn Methodist Hospital, Brooklyn, NY 11215, USA

**Keywords:** breast cancer, cell-of-origin, histone deacetylase, niclosamide, drug repurposing, heat shock protein

## Abstract

**Simple Summary:**

The human body is composed of hundreds of different normal cell types. Some of the important and unique features of these normal cell types are inherited by the cancer cells that arise from them. Indeed, the activities of the tumor gene mutations are constrained by this normal cell inheritance. For example, we previously found that the same oncogenes produce highly malignant tumors in some cell types but not in others. In this study, we demonstrate that this cell-of-origin difference can be used to develop cell-targeted therapies, a novel concept that differs from gene-targeted therapies. This cell-based approach guided us to a surprising discovery that a drug approved for treating tapeworm infestation in combination with an antibiotic derivative can significantly enhance the killing of breast cancer cells. Since these drugs are already approved for clinical use, it may be possible to repurpose them to treat breast cancer.

**Abstract:**

We recently identified a cell-of-origin-specific mRNA signature associated with metastasis and poor outcome in triple-negative carcinoma (TNBC). This TNBC cell-of-origin signature is associated with the over-expression of histone deacetylases and zinc finger protein HDAC1, HDAC7, and ZNF92, respectively. Based on this signature, we discovered that the combination of three drugs (an HDAC inhibitor, an anti-helminthic Niclosamide, and an antibiotic Tanespimycin that inhibits HSP90) synergistically reduces the proliferation of the twelve tested TNBC cell lines. Additionally, we discovered that four out of five inflammatory breast carcinoma cell lines are sensitive to this combination. Significantly, the concentration of the drugs that are used in these experiments are within or below clinically achievable dose, and the synergistic activity only emerged when all three drugs were combined. Our results suggest that HDAC and HSP90 inhibitors combined with the tapeworm drug Niclosamide can achieve remarkably synergistic inhibition of TNBC and IBC. Since Niclosamide, HDAC, and HSP90 inhibitors were approved for clinical use for other cancer types, it may be possible to repurpose their combination for TNBC and IBC.

## 1. Introduction

Breast cancer is one of the most prevalent malignancies, with an estimated 2.3 million new cases and 685,000 deaths per year globally. Approximately 60 to 70% of breast cancers express the estrogen receptor (ER+), 10 to 15% are ERBB2-amplified (HER2+), and 10 to 15% are triple-negative breast cancers (TNBC) that are ER-, PR-, and Her2-. Unfortunately, while there are targeted therapies available for ER+ and HER2+ breast cancer, no targeted therapy is available for TNBC or IBC (Inflammatory Breast Cancer), which have a worse outcome than other subtypes.

The major cause of death in all subtypes of breast cancer is the metastatic spread of the cancer cells through lymphatics and blood vessels to other organs. A very aggressive and lethal clinical type of cancer, named inflammatory breast cancer (IBC), is more common in Black/African American, younger than 40, overweight patients. IBC is associated with extensive dermal lymphovascular invasion (LVI), and high metastatic risk. It is worth mentioning that the term pseudo-inflammatory may have been more accurate for this entity; because the edema, erythema, and tenderness mimicking breast inflammation (mastitis) is due to dermal tumor emboli plugging lymphovascular circulation rather than true inflammation. Nevertheless, IBC is a very difficult challenge in the clinic; despite the distinct biology of IBC, there is no specific therapy for this disease. Patients with IBC are treated with systemic treatments similar to other non-inflammatory breast cancers [1].

The example of breast cancer suggests that targeted therapies can leverage acquired targets (HER2 amplification) and intrinsic targets (ER expression). The breast cancer-acquired amplification of ERBB2 has been targeted with anti-HER2 antibodies, biologics, and small-molecule ERBB2 kinase inhibitors. In the case of hormonally driven breast cancer, the overexpression of ER seems to reflect the normal cell-of-origin or differentiation lineage since ER mutations or amplifications are exceedingly rare in untreated patients. In some cases, ER mutations emerge after anti-estrogen treatment as a resistance mechanism. Nevertheless, the anti-estrogens that block ER transcriptional activity or suppress estrogen production have been highly effective in ER+ breast cancer.

**Figure 1:**
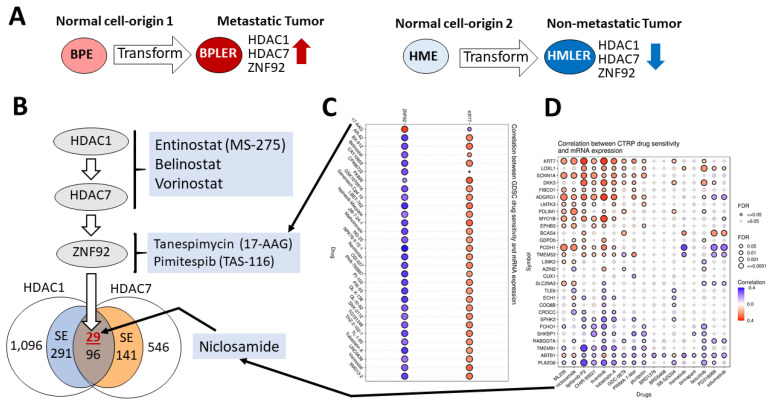
Identification of metastatic TNBC cell-of-origin targets and drugs. (**A**) The estrogen receptor negative normal breast cells cultures, BPE and HME, were generated from the same donor [2]. Transformation of BPE and HME with the same genes gave rise to metastatic BPLER and non-metastatic HMLER tumor cell lines with a TNBC phenotype [2]. The metastatic TNBC BPLER cells are associated with higher expression of HDAC1, HDAC7, and ZNF92 compared to non-metastatic TNBC HMLER cells [25,27]. (**B**) HDAC1 upregulates HDAC7, which in turn upregulates 29 super-enhancer (SE) associated genes with ZNF92 binding sites [27]. This HDAC1-HDAC7-ZNF92 axis can be targeted by HDAC inhibitors (Entinostat, Belinostat, and Vorinostat). (**C**) ZNF92 expression correlates only with 17-AAG sensitivity (arrow) among 265 small molecules in the Genomics of Drug Sensitivity in Cancer (GDSC) dataset, with IC50 in 860 cell lines and their corresponding mRNA gene expression (red circle, top left). All the other drugs negatively correlate with ZNF92 (blue circles). GSCA only draws plots for the top 30 ranked drugs; blue-filled circles represent negative correlation, red-filled circles represent positive correlation, the color intensity reflects higher correlation, the circle size is positively correlated with the false discovery rate (FDR) significance, and the black outline border indicates FDR ≤ 0.05. See supplemental data for details. (**D**) The correlation of the 29 gene ZNF92 signature with the IC50 of 481 small molecules in 1001 cell lines and corresponding mRNA gene expression from Genomics of Therapeutics Response Portal (CTRP). The mRNA expression data and drug sensitivity data were merged. Pearson correlation analysis was performed to get the correlation between gene mRNA expression and drug IC50. *p*-value was adjusted by FDR. Among the top ranked drugs shown, Niclosamide was ranked #2 (arrow). The blue-filled circles represent a negative correlation, red-filled circles represent a positive correlation, the color intensity reflects a higher correlation, the circle size is positively correlated with the false discovery rate (FDR) significance, and the black outline border indicates FDR ≤ 0.05. See supplemental data for details.

Based on clinical observations, we hypothesized that the intrinsic properties of the normal cell-of-origin might be involved in shaping tumor biology in other subgroups of breast cancer, such as TNBC [2]. We used identical genetic elements to test this hypothesis to transform two different ER-negative normal breast cell-origins isolated from the same donor [2]. While one normal cell-origin developed invasive and metastatic TNBC, the other normal cell-origin developed non-metastatic indolent TNBC, confirming our hypothesis [2] (Figure 1A). Since then, other investigators independently verified our hypothesis in numerous cancer types [3,4,5,6,7,8,9,10,11,12,13,14,15]. However, these cell-origin signatures have been challenging to translate into mechanistic insights because they involve thousands of genes [16,17,18]. As such, we had a similar challenge, having identified more than 6000 breast cell-origin-associated mRNAs in our metastatic TNBC model.

We reasoned that examining histone modifications could potentially uncover a mechanism that may propagate the normal cell-origin signature in TNBC [19]. After exploring several candidates [20,21,22,23,24], we found that HDAC7 and HDAC1 were specifically upregulated in the metastatic TNBC cell lineage we developed but not in the isogenic non-metastatic cell lineage [25]. Next, we determined that in these cells, HDAC1 is upstream of HDAC7, and these two HDACs co-regulate 1243 mRNAs in the metastatic TNBC cells [26]. Finally, we found that approximately 10% of the HDAC1&7 upregulated mRNAs (*n* = 125) are increased via super-enhancers in metastatic TNBC [27], but not in non-metastatic TNBC [25]. Further, a 60 gene mRNA subset of this cell-origin signature predicted remarkably shorter breast cancer survival, 8.7 years overall and 6.2 years relapse-free, respectively [26].

Next, we discovered that twenty-nine genes that are co-upregulated by HDAC1&7 in metastatic TNBC have a ZNF92 binding site in their promoter [26]. ZNF92 is a primate-specific member of the KRAB-ZNF (Kruppel-associated box domain zinc finger) family with more than 700 genes encoding tissue-specific transcription suppressors. ZNFs have been an understudied protein family (534 papers in PubMed for 700 genes). Even among this family, ZNF92 is an exceptionally unexplored protein since its cloning in 1991, as it was only mentioned once among 121 genes that are altered with cholesterol-lowering drug atorvastatin in the liver cell line HepG2 [28]. Previous to our study, ZNF92 had not been examined in any cancer type.

We inferred that the metastatic TNBC cell-origin-related HDAC1-HDAC7-ZNF92 axis could be targeted using a combination of small molecules. As a proof-of-concept, we report in this study that combining an anti-worm drug, an antibiotic that inhibits HSP90, and a histone deacetylase inhibitor can synergistically inhibit the proliferation of sixteen out of seventeen TNBC and IBC cell lines (Figure 1B). The repurposing of these clinically approved drugs provides an opportunity to develop a cell-targeted therapy for TNBC and IBC.

## 2. Materials and Methods

Reagents: Entinostat (Active Motif 14043, Carlsbad, CA, USA), Vorinostat (Abcam-ab1444480, Cambridge, MA, USA), Belinostat (Tokyo Chemical Industry-B5888, Chou-Ku, Tokyo, Japan), Niclosamide (Bio-Vision-1826, Milpitas, CA, USA) [25,26,27]. Tanespimycin/17AAG (Bio-Vision-1774, Milpitas, CA, USA) and Pimitespib (Tas1116, Cayman Chemical Company-33779, Ann Arbor, MI, USA) were reconstituted in DMSO (Sigma Aldrich D-8418, St. Louis, MO, USA). Cell Proliferation Reagent WST-1 (11644807001, Sigma Aldrich, St. Louis, MO, USA).

Cell culture: The ATCC sourced breast cancer cell lines were previously acquired and extensively validated including STR profiling in our previous studies [11,25,27,29]. All the ATCC cell lines, including HCC38, HCC202, HCC1187, HCC 1937, HCC 1143, HCC 1954, MFM223, CAL148, MDAMB231, SKBR3, ZR75-1, ZR75-B, MCF7, MDA-MB-231, MDA-MB-435, BT549, BT20, and T47D were maintained in their respective media as recommended by ATCC (Gibco, Thermo-Fisher, NY, USA). The inflammatory breast cancer cell lines IBC02 (FC-IBC-02) IBC03, KPL4, Sum149, and Sum190 were generously provided by Dr. Sandra Fernandez and cultured in an F-12 medium (Gibco 11765-054, Brooklyn, NY, USA). All the drug sensitivity experiments were carried out in a medium supplemented with 2% heat-inactivated fetal bovine serum. The BPLER and HMLER cells were previously characterized, deposited to the American Type Culture Collection and the European Collection of Authenticated Cell Cultures (ATCC # CRL-3546 and ECACC # 20012032 and 20012041), and cultured in BMI-T medium (US Biological, cat# 506387.500, Salem, MA, USA) [27]. All the cells were cultured at 37 °C and 5% CO_2_ in a humidified incubator.

WST cell proliferation assay: The changes in cell proliferation and viability due to treatment with Entinostat, Vorinostat, Belinostat, Niclosamide, Tanespimycin, and Pimitespib alone or in combination were determined by WST assay following manufacturer’s protocol. Briefly, ~3000 cells per well were plated in 96-well plates and treated with various drugs for 7 days. At the end of the specified time following drug treatment, the WST reagent (10 µL) was added into each well, incubated at 37 °C for 2 h, and the color absorbance of each well was recorded at 450 nm with a Thermo Labsystems Multiskan Ascent microplate reader (Pittsburgh, PA, USA) with a blank reference.

Drug screening: The Gene Set Cancer Analysis (GSCA) and the Cancer Therapeutics Response Portal (CTRP) online analysis tools were used to identify drugs that may target ZNF92 and its 29 gene-signature. These platforms integrate more than 10,000 comprehensive genomic data sets of 33 diverse types of cancers from TCGA with 750 drug molecules [30,31,32].

Graphical representation and Statistical analyses: GraphPad Prism (V9) software was used for statistical analysis and graph generation. All data are expressed as the mean ± SD of six independent experiments. The differences between the control and the treatment groups were determined by one-way ANOVA, and significance was determined by using Dunnett’s multiple comparison test (*p* < 0.01).

## 3. Results

We utilized the Gene Set Cancer Analysis (GSCA) online platform [30] to discover that ZNF92 expression is uniquely associated with sensitivity to the 90-kDa heat shock protein (Hsp90) inhibitor Tanespimycin (17-AAG), a synthetic analog of the antibiotic Geldanamycin [33]. HSP90 is a molecular chaperone that regulates protein hemostasis by regulating the folding, stabilization, activation, and degradation of over 400 proteins [34].

The GSCA includes the Genomics of Drug Sensitivity in Cancer (GDSC) dataset that contains IC50 of 265 small molecules in 860 cell lines and their corresponding mRNA expression [31]. Significantly, 17-AAG was the only hit for ZNF92 in the GDSC, as all the other top 30 hits are anti-correlated, meaning that ZNF92 expression predicted resistance to these drugs (Figure 1C).

Using the Genomics of Therapeutics Response Portal (CTRP) [30,32], we discovered that the 29 gene mRNA signature downstream of the HDAC1-HDAC7-ZNF92 axis correlates with net sensitivity to 17/481 small molecules in 1001 cell lines (Figure 1D). The top five CTRP hits in rank order include ML239 (breast cancer stem cell inhibitor), Niclosamide (anti tapeworm drug), Tipifarnib (H-Ras inhibitor), CHIR-99021 (GSK-3α/β inhibitor), and Tivantinib (MET inhibitor). Among these, only two were approved by the U.S. Food and Drug Administration (FDA); Niclosamide was approved by the FDA to treat tapeworm infection in 1982 [35], and Tipifarnib recently received a breakthrough therapy designation by the FDA for the treatment of patients with recurrent or metastatic HRAS mutant head and neck squamous cell carcinoma (HNSCC) [36]. Since Niclosamide was the highest-ranked FDA approved drug, we selected this compound for the combination experiments.

**Figure 2:**
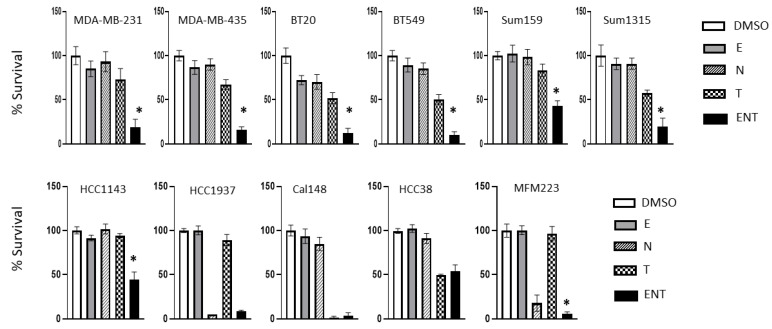
Triple-negative breast carcinoma; the combination of HDAC-i Entinostat (E), Niclosamide (N), and HSP90-i Tanespimycin (T). The TNBC cell lines BT549, BT20, HCC38, CAL148, HCC 1143, HCC 1913, MDA-MB-231, MDA-MB-435, MFM223, Sum159, and Sum1315 are treated with Entinostat (E: 100 nM), Niclosamide (N: 100 nM), and Tanespimycin (T: 50 nM) alone and in combination (ENT) in 96-well plates for 7 days with indicated drugs. The inhibition of cell proliferation by E, N, T, and ENT is measured by WST assay. The relative cell number is calculated compared to vehicle control (DMSO) and expressed as mean ± SD (*n* = 6) of percent viability. (*) Inhibition in cell proliferation is greater than expected by additivity. See Supplementary Table S1 for details.

As we have previously shown that Entinostat (MS-275) inhibits both HDAC1 and HDAC7 [25], we reasoned that combining Entinostat (E) with Tanespimycin (T) and Niclosamide (N) may target all the components of the HDAC1-HDAC7-ZNF92 axis. First, we carried out dose–response experiments in several TNBC cell lines and determined the drug concentrations that have minimal effect on cell proliferation (Figure S1A–C). Encouragingly, while these low doses reduced cell proliferation only by 16% individually, the three-drug combination resulted in over 84% average inhibition of proliferation, consistent with a synergistic activity (Figure S1D). We confirmed these results in multiple TNBC cell lines, where the observed average inhibition was significantly greater than expected by additive activity (Figure 2 and Table S1). Furthermore, in several TNBC cell lines, there was exceptional sensitivity to Niclosamide (HCC1913, MFM223) or Tanespimycin (Cal148) (Figure 2).

These results provided a proof-of-concept that HDAC and HSP90 inhibitors can be combined with a tape-worm drug Niclosamide to achieve synergistic effects in TNBC. However, this drug combination presented two challenges for immediate clinical translation; Entinostat is still undergoing Phase II–III trials and has not been FDA approved. Moreover, even though Tanespimycin concentrations required for activity in preclinical models could be safely achieved in patient plasma in phase 1/2 clinical trials, it was reported that the manufacturer of Tanespimycin declined to advance it to phase III trials.

To potentially translate our findings to the clinic more rapidly, we searched for clinically approved alternatives for Entinostat and Tanespimycin. Currently, there are five FDA-approved HDAC inhibitors (HDAC-i) available for cancer treatment: Vorinostat (SAHA), Belinostat (PXD-101), Romidepsin (FK-228), Epidaza (HBI-8000), and Panobinostat (LBH589) [37]. Furthermore, a new orally available HSP90 inhibitor, TAS-116 (Pimitespib), was recently approved to treat gastrointestinal stromal tumors that have progressed after chemotherapy in Japan [38].

By way of single drug dose–response experiments we determined the minimally effective doses of Pimitespib (P), Vorinostat (V), and Belinostat (B) (Figure S2A). First, we found that it was possible to replace one HSP90 inhibitor (HSP90-i) Tanespimycin with another HSP90-i (Pimitespib) in combination with Entinostat and Niclosamide with similar synergistic results (Figure S2B). Next, we showed that HDAC inhibitor Entinostat could be replaced by two different HDAC-i Vorinostat (V) and Belinostat (B) in combination with Niclosamide (N) and Pimitespib (P) in five out of five TNBC cell lines tested. In these experiments, we expected approximately 20 to 30% inhibition of cell proliferation if these three drugs acted additively. In contrast, we observed approximately 70 to 80% inhibition consistent with synergistic action (Figure S3). The ability to swap different small molecules for the same target suggests that the observed synergies are probably due to bona fide target synergies and not the result of an idiosyncratic drug interaction.

**Figure 3:**
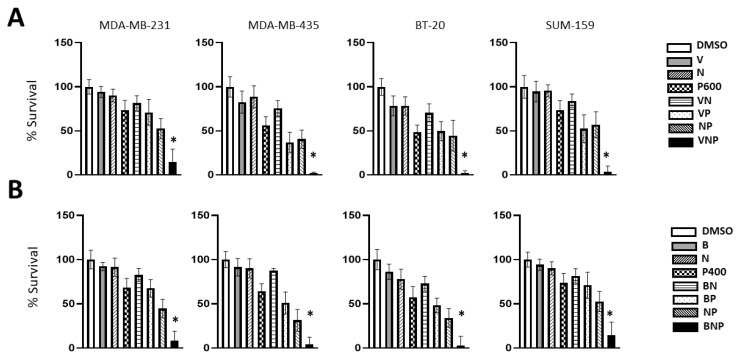
Triple-negative breast carcinoma; the combination of HDAC-i Vorinostat or Belinostat (B), with Niclosamide (N) and HSP90-i Pimitespib (P). The TNBC cell lines BT20, MDA-MB-231, MDA-MB-435 and Sum159 are cultured in 96-well plates for 7 days with indicated drugs. The inhibition of cell proliferation is measured by WST assay. The relative cell numbers are calculated compared to vehicle control (DMSO) and expressed as mean ± SD (*n* = 6) of percent viability. (**A**) Vorinostat (V: 1000 nM), Niclosamide (N: 100 nM), and Pimitespib (P: 600 nM), and (**B**) Belinostat (B: 1000 nM), Niclosamide (N: 100 nM), and Pimitespib (P: 400 nM). (*) Inhibition in cell proliferation is greater than expected by additivity. See Supplementary Table S1 for details.

Next, we determined the lowest combined doses that can produce near complete inhibition of TNBC proliferation and determined that 400–600 nM Pimitespib combined with 100 nM Niclosamide and 1000 nM Vorinostat produced 96–98% inhibition of proliferation in three out of four TNBC cell lines (Figure 3A), which was replicated with another HDAC inhibitor Belinostat (Figure 3B). It is worth pointing out that 10 to 200-fold higher concentrations of Vorinostat, Belinostat, Pimitespib and Niclosamide are achievable in the clinic (Table 1) [38,39,40,41,42,43,44,45]. Therefore, it may be possible to eliminate tumor cells with a three-drug combination at clinically relevant doses.

Lastly, we previously showed that HDAC inhibitors inhibited the self-renewal of IBC tumor spheroids and tumor emboli [46,47]. Significantly, 4/5 IBC cell lines (IBC02, IBC03, KPL4, and SUM-149) were found to be sensitive to both EPN and BPN triple-drug combinations (Figure S4 and Table S2).

## 4. Discussion

Historically, three or more drugs have been routinely combined for many cancer treatments [48]. Partly due to the difficulty of screening high-order combinations, new drug candidates are often tested in combination with first-line treatments, sometimes without a compelling hypothesis [49,50,51,52,53,54,55]. As expected, most of the 54 breast clinical trials with HDAC and HSP90 inhibitors have been in combination with anti-estrogen, anti-HER2 and/or chemotherapy drugs. We have not found any breast cancer trials for niclosamide (Table S3).

However, these combinations do not necessarily originate with high-order experimental synergy screens, which are currently unfeasible due to the exponential increase in complexity to analyze the interaction of more than two drugs [56]. For example, the Gene Set Cancer Analysis (GSCA) we used in this study integrates >10,000 genomic data sets and 750 drugs from GDSC and CTRP, representing more than 70 million possibilities for 3-drug combinations. Thus, a systematic three-drug biological screen was not feasible for us to even with 20 candidates, because it would require testing 1140 three-drug combinations [56] (Figure 1). As a solution, it is thought that pairwise interaction scores can provide reliable estimates for three-drug interactions [57]. However, in our case, the pairwise results of HDAC-i, HSP90-i and Niclosamide were not significantly additive. Hence, we assign import to the de novo discovery of HDAC-i, HSP90-i, and Niclosamide synergy, which would not have been discovered empirically without a cell-of-origin hypothesis-based approach [58].

In order to illustrate the three-drug synergetic effect, we purposefully used low concentrations of HDAC-i, HSP90-i, and Niclosamide in our experiments. Nevertheless, we observed up to 98% reduction in TNBC cell proliferation, suggesting that it might be possible to achieve meaningful results with clinically recommended doses of Niclosamide (2000 mg/day, 220 mg/m^2^) [35,59], Vorinostat (400 mg) [60], Pimitespib (160 mg/day) [61], and Belinostat (1000 mg/m^2^/day) [43,52]. At these doses, the side effects of HDAC-i include diarrhea, fatigue, pyrexia, nausea, thrombocytopenia, and anorexia. While Niclosamide side effects are generally non-overlapping (itching, drowsiness, dizziness, and skin rash); the side effects of HSP90 inhibitor Pimitespib partially overlap with HDAC-i (diarrhea, decreased appetite, malaise, renal impairment, and anemia). Whether any of these toxicities would be exacerbated or other toxicities would emerge with a three-drug combination remains to be seen. However, in our experiments, we used Entinostat (100 nM), Niclosamide (100 nM), Tanespimycin (50 nM), Belinostat (1 uM), Vorinostat (1 uM), and Pimitespib (0.2–0.6 uM) at doses that are 10 to 1000-fold lower than the peak serum concentrations achieved in different clinical trials for Entinostat (1 uM), Niclosamide (2 uM), Tanespimycin (15 uM), Belinostat (200 uM), Vorinostat (40 M), and Pimitespib (7 uM). Therefore, it may be possible to manage these side effects by lowering the dose of some drugs when used in combination. Moreover, two recent studies reported the development of novel dual HDAC and HSP90 inhibitors, where a single small molecule inhibited both HDAC and HSP90 that were tested on age-related macular degeneration [62] and leukemia [63].

The targeted therapy concept has often been mentioned for a class of drugs that target tumor-specific gene alterations. While these gene-targeted therapies have been transformative for HER2+ breast cancer, other breast cancer subtypes do not necessarily present such a straightforward gene-targeted therapy opportunity due to heterogeneity and clonal evolution.

It was recently reported that the cell-of-origin patterns dominate the molecular classification of 10,000 tumors from 33 types of cancer [64]. We expanded the concept of cell-of-origin to targeted therapy because a substantial portion of the normal cell-origin mRNA, protein, and DNA methylation signatures are retained in breast cancer [2,11,12,65,66]. These cell-origin signatures can be associated with clinically relevant tumor features such as metastasis [2,26]. Thus, we postulated that such a persistent cell-origin signature could be targeted with drug combinations. It is worth mentioning that the HDAC1-HDAC7-ZNF92 axis and their 29 downstream targets are rarely mutated in human breast cancers, consistent with a cell-of-origin signature (Figure S5 and Table S4). As a proof-of-concept, we show that this metastatic breast cancer cell-of-origin signature can be synergistically targeted with HDAC and HSP90 inhibitors combined with Niclosamide in sixteen out of seventeen breast cancer cell lines.

## 5. Conclusions

Despite their expression ubiquitously in all tissues, hereditary mutations cause tissue-specific cancers [67]. It seems that rather than being a blank canvas, the tissue origin is an active partner in the development of familial tumors [68]. Conversely, because the mutational spectrum of sporadic tumors seems less tissue-specific, it has been proposed that sporadic tumors should be classified according to molecular alterations regardless of their tissue origin [68]. However, recent basket trials suggest that the response to targeted anti-cancer drug response often depends on the tissue type [68]. Consistent with these observations, our work suggests that the cell-of-origin is involved in shaping breast tumor phenotype and drug response [69]. Further, we demonstrate that the cell-of-origin signature can be leveraged to develop cell-targeted therapies.

## Figures and Tables

**Table 1:** The comparison of the peak serum concentrations achieved for HDAC inhibitors, HSP90 inhibitors, Niclosamide, and the drug concentrations used in this study.

Drug	Phase I–II C_max_ (nM)	Experimental Three-Drug (nM)	Ratio
Entinostat	1000	100	10
Vorinostat	40,000	1000	40
Belinostat	200,000	1000	200
Niclosamide	2000	100	20
Tanespimycin	15,000	50	300
Pimitespib	7000	200–600	11–35

## Data Availability

The data supporting the results can be found at GSCA: Gene Set Cancer Analysis (hust.edu.cn) and cBioPortal https://www.cbioportal.org/.

## Supplementary Materials

The following supporting information can be downloaded at: https://www.mdpi.com/article/10.3390/cancers15020332/s1, Figure S1: Triple-negative breast carcinoma dose–response and test combination of HDAC inhibitor Entinostat (E), Niclosamide (N), and HSP90 inhibitor Tanespimycin (T). Figure S2: HSP90 inhibitor Pimitespib (P) and HDAC inhibitors Vorinostat (V) and Belinostat (B). Figure S3: Triple-negative breast carcinoma triple-drug combinations. Figure S4: Inflammatory breast cancer triple-drug combinations. Figure S5: Frequency of genomic alterations in the cell-of-origin signature. Table S1: Survival of TNBC cells with HDAC-i, HSP90-i, and niclosamide. Table S2: Survival of inflammatory breast cancer cells with HDAC-i, HSP90-i, and niclosamide. Table S3: Breast cancer clinical trials with HDAC and HSP90 inhibitors. Table S4: The frequency of genomic alterations in the cell-of-origin signature
