# Supplementary material for: Cell-of-Origin Targeted Drug Repurposing for Triple-Negative and Inflammatory Breast Carcinoma with HDAC and HSP90 Inhibitors Combined with Niclosamide
Source: Cancers (Basel). 2023 Jan 4;15(2):332. doi: 10.3390/cancers15020332 (PMC9856736; doi:10.3390/cancers15020332)
Supplement: Supplementary file 1 [file cancers-15-00332-s001.zip › Supplementary Material.zip!/Supplementary Material.pdf]

Supplementary Material

Cell-of-origin targeted drug repurposing for triple-negative and inflammatory breast carcinoma with HDAC and HSP90 inhibitors combined with niclosamide

Authors: Udayan Bhattacharya[1,*], Mohammad Kamran[1,*], Marwa Manai[2], Massimo Cristofanilli[2] and Tan A. Ince[1, 3, ‡]

(1) Weill Cornell Medicine, Department of Pathology and Laboratory Medicine (2) Weill Cornell Medicine, Division of Hematology-Oncology and (3) New York Presbyterian, Brooklyn Methodist Hospital. The first two authors contributed equally (*).

(‡) Corresponding author, contact: tai9015@med.cornell.edu

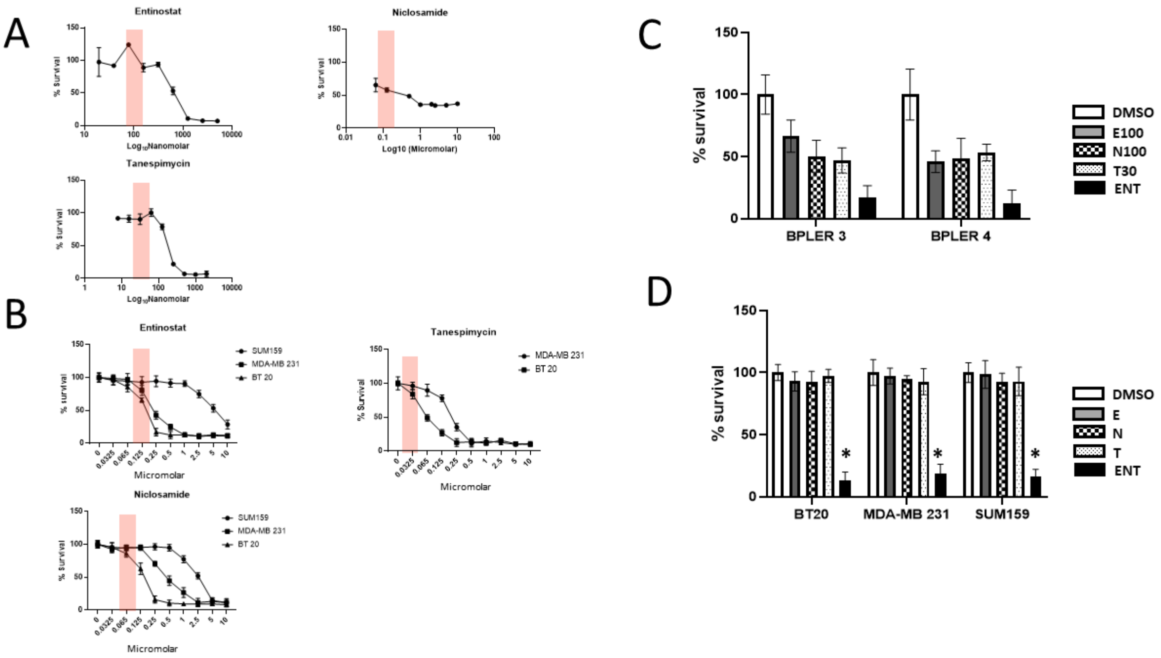

Supplementary Figure S1. Triple-negative breast carcinoma dose response and test combination of HDAC inhibitor Entinostat (E), Niclosamide (N), and HSP90 Tanespimycin (T).

All experiments were performed in 96-well plates for 7 days. DMSO was used as vehicle control. Inhibition of cell proliferation is measured by the WST assay. Each value is expressed as mean ± SD ($n = 6$) of percent viability. (*) Inhibition in cell proliferation greater than expected by additivity. See supplementary table 1 for details.

A) Dose response plots of Entinostat (E), Niclosamide (N) and Tanespimycin (T) in BPLER-TNBC cells (0, 15, 32.5, 65, 125, 250, 500, 1000, 2500, 5000,10000 nM).

B) Dose response plots of Entinostat (E), Niclosamide (N) and Tanespimycin (T) in BT20, MDAMB231 and Sum159 TNBC cells lines (0, 15, 32.5, 65, 125, 250, 500, 1000, 2500, 5000,10000 nM). Highlighted regions in the graphs show the range of doses used for three-drug synergy experiments.

C) Three drug combination of Entinostat (E: 100 nM), Niclosamide (N: 100 nM) and Tanespimycin (T: 30 nM) in BLER cells.

D) Three drug combination of Entinostat (250 nM: Sum159, 100nM: MDA-MB231, 50nM: BT20), Niclosamide (250 nM: Sum159, 100 nM: MDA-MB231, 50 nM: BT20), and Tanespimycin (50 nM: Sum159 and MDA-MB231, 30nM:BT20).

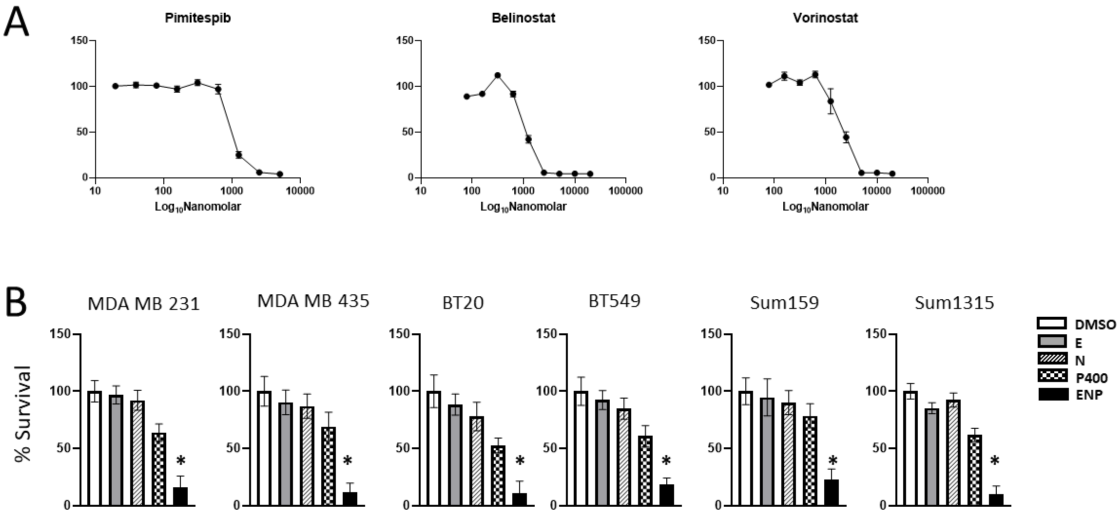

Supplementary Figure S2. HSP90 inhibitor Pimitespib (P) and HDAC inhibitors Vorinostat (V) and Belinostat (B)

A) Dose response plots of HSP90 inhibitor Pimitespib (P) and HDAC inhibitors Vorinostat (V) and Belinostat (B) in BPLER-TNBC cells (0, 15, 32.5, 65, 125, 250, 500, 1000, 2500, 5000, and 10000 nM).

B) Three drug combination of Entinostat (50nM for BT20 and 100 nM for all other cell lines), Niclosamide (N: 100nM), and Pimitespib (P: 400nM).

The cells are cultured in 96-well plates for 7 days with drug. Inhibition of cell proliferation is measured by WST assay, the relative cell numbers are calculated compared to vehicle control (DMSO) and expressed as mean ± SD (*n* = 6) of percent viability. (*) Inhibition in cell proliferation greater than expected by additivity. See supplementary table 1 for details.

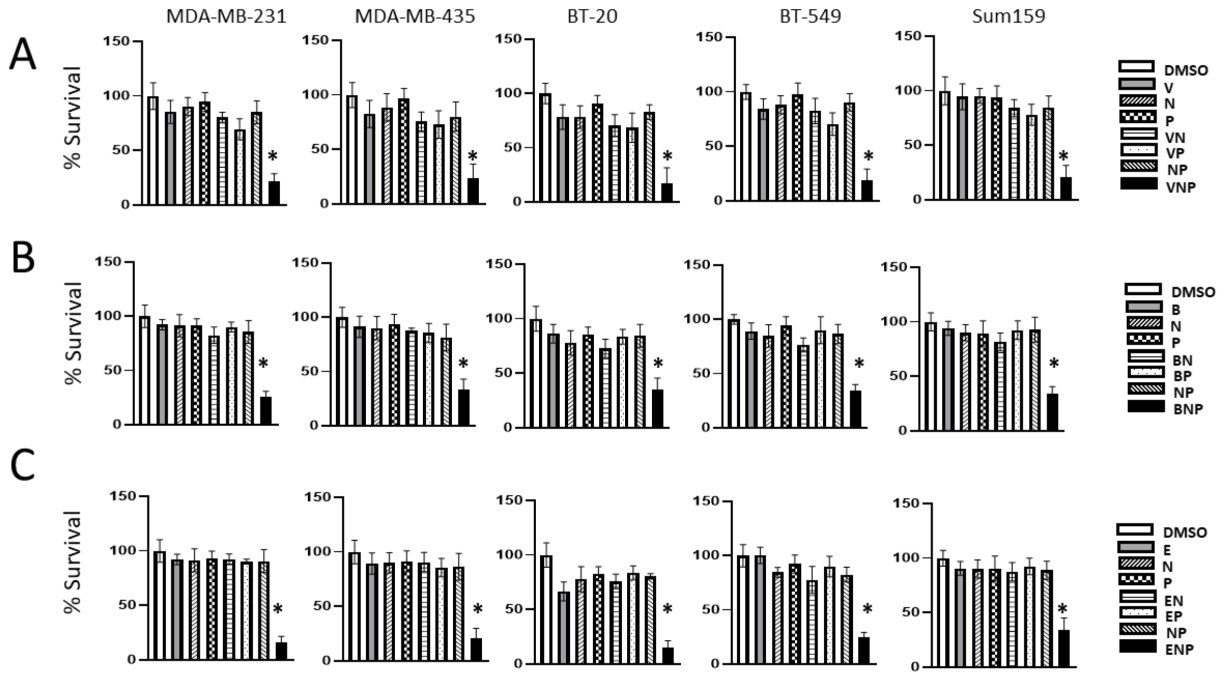

Supplementary Figure S3. Triple-negative breast carcinoma triple-drug combinations

Combination of HDAC inhibitor Vorinostat (V), Belinostat (B) or Entinostat (E), with Niclosamide (N) and HSP90-I Pimitespib (P). The TNBC cell lines BT20, MDAMB231, MDAMB435 and Sum159 are cultured in 96-well plates for 7 days with drug. Inhibition of cell proliferation is measured by WST assay, the relative cell numbers are calculated compared to vehicle control (DMSO) and expressed as mean ± SD (*n* = 6) of percent viability. (*) Inhibition in cell proliferation greater than expected by additivity. See supplementary table 1 for details.

A) Vorinostat (V: 1000 nM), Niclosamide (N: 100 nM), and Pimitespib (P: 200 nM).

B) Belinostat (B: 1000 nM), Niclosamide (N: 100 nM), and Pimitespib (P: 200 nM).

C) Entinostat (E: 100 nM), Niclosamide (N: 100 nM), and Pimitespib (P: 200 nM).

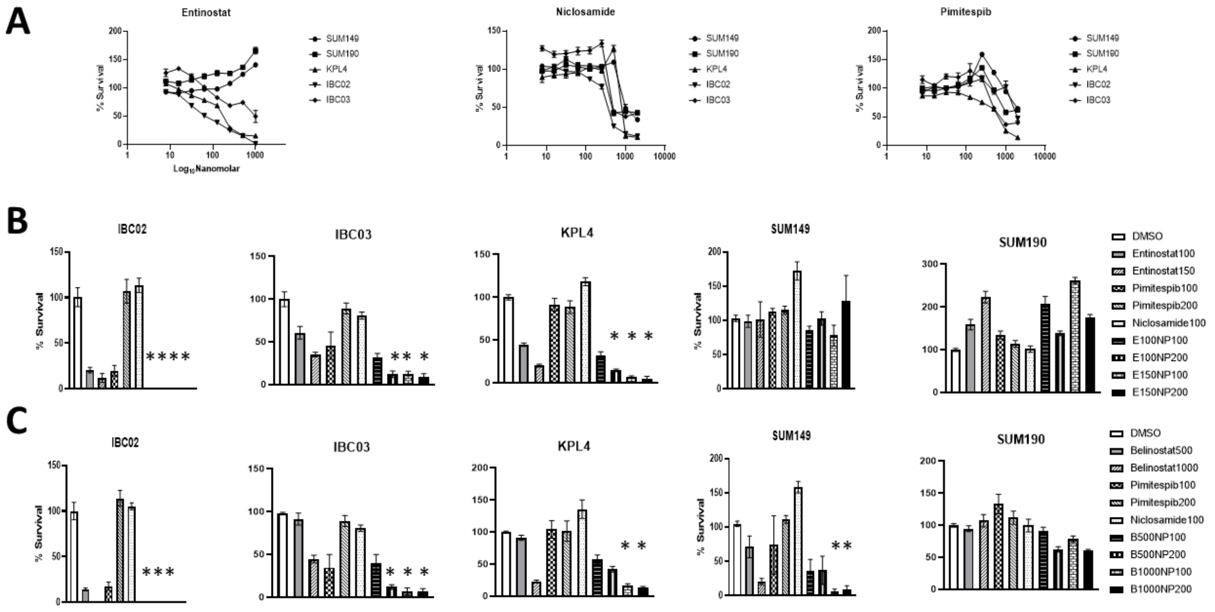

Supplementary Figure S4. Inflammatory breast cancer triple-drug combinations

The combination of HDAC inhibitor Entinostat (E) and Belinostat (B) with Niclosamide (N) and HSP90 inhibitor Pimitespib (P) in inflammatory breast cancer (IBC) cell lines. The IBC03, SUM-149 and SUM-190 cell lines are cultured in F12 medium (Gibco 11765-054) and KPL-4 is cultured in Ham's F12 medium (Corning 10-080-CV) with the indicated drugs. The inhibition of cell proliferation is measured by WST assay, the relative cell numbers are calculated compared to vehicle control (DMSO) and expressed as mean ± SD (n = 6) of percent viability. (*) Inhibition in cell proliferation greater than expected by additivity. See supplementary table 2 for details.

A) Dose response plots of HDAC inhibitor Entinostat (E), Niclosamide (N) and Pimitespib (P) in IBC cell lines (0, 15, 32.5, 65, 125, 250, 500, 1000, 2500, 5000, and 10000 nM).

B) Entinostat (100 or 150 nM), alone or combined with Pimitespib (100 or 200 nM), and Niclosamide (100 nM).

C) Belinostat (500 or 1000 nM), alone or combined with Pimitespib (100 or 200 nM), and Niclosamide (100 nM).

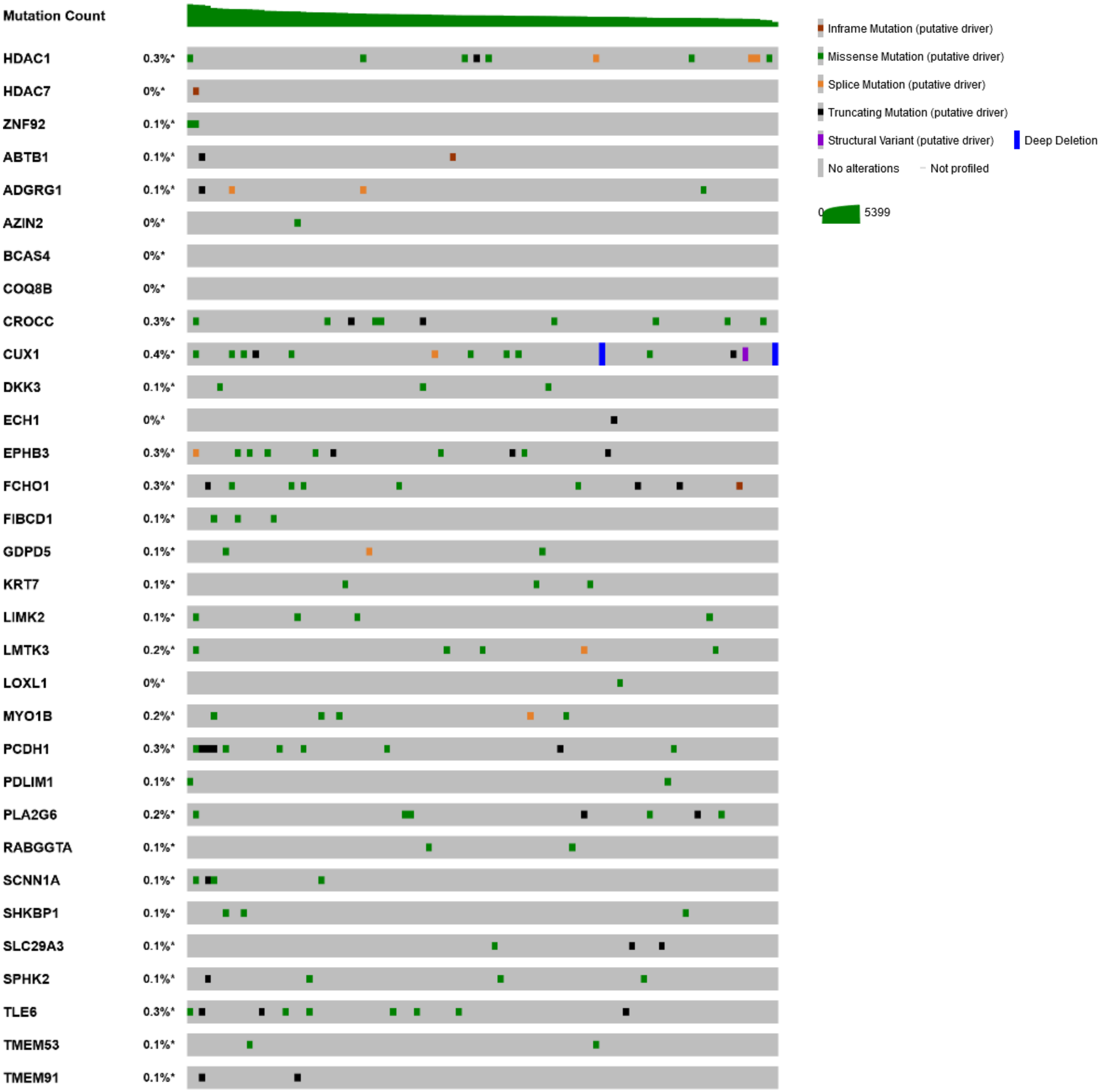

Supplementary Figure S5. Frequency of genomic alterations in the cell-of-origin signature

The image illustrates that frequency of mutations, structural variants and copy number changes in HDAC1, HDAC7, ZNF92 and the 29 genes with the ZNF92 binding sites (excluding alterations of unknown significance). The analysis is carried out in the cBioPortal combined TCGA and Metabric datasets with 3,593 breast cancer samples and shows that none of the genes in the cell-origin associated HDAC1-HDAC7-ZNF92 axis and their 29 downstream targets have *bona fide* driver mutations in more than 0.4% of breast cancer (https://www.cbioportal.org/)[1, 2]

Figure 2		MDA-MB231	MDA-MB435	BT 20	BT549	Sum 159	Sum-1315	Average
	DMSO	100	100	100	100	100	100	100.0
	E	85.2	86.7	92.3	89.4	102.4	90.7	91.1
	N	93.2	90.1	70.2	85.2	98.4	90.9	88.0
	T	72.8	67.3	51.8	50.5	83.6	57.5	63.9
Observed	ENT	19.2	15.8	12.3	10.3	42.9	19.7	20.0
Expected additive		57.8	52.6	33.6	38.5	84.2	47.4	52.3

Figure 3A		MDA-MB231	MDA-MB435	BT 20	Sum - 159	Average	Figure 3B	MDA-MB231	MDA-MB435	BT 20	Sum - 159	Average
	DMSO	100	100	100	100	100.0	DMSO	100	100	100	100	100.0
	V	94.2	82.6	78.3	94.6	87.4	B	92.3	91.2	86.3	94.2	91.0
	N	90.1	88.7	78.2	95.4	88.1	N	91.4	90.1	77.9	90.1	87.4
	P	73.6	56.4	48.7	73.4	63.0	P	68.4	64.1	57.2	73.6	65.8
	VN	81.6	75.6	70.4	84.1	77.9	BN	82.6	87.8	72.8	81.6	81.2
	VP	70.9	36.7	49.7	52.4	52.4	BP	67.6	50.9	48.2	70.9	59.4
	NP	52.6	40.5	44.6	56.9	48.7	NP	44.5	31.4	34.1	52.6	40.7
Observed	VNP	14.3	1.8	1.9	3.8	5.5	BNP	8.6	3.9	2.6	14.3	7.4
Expected additive		62.5	41.3	29.8	66.2	50.0		57.7	52.7	38.5	62.5	52.8

Supplementary Figure S1		MDA-MB231	BT 20	Sum - 159	Average
	DMSO	100	100	100	100
	E	97.2	92.94	98.4	96.2
	N	94.8	92.5	92.7	93.3
	T	92.6	97.3	92.8	94.2
Observed	ENT	18.8	12.9	16.3	16
Expected additive		85.3	83.6	84.6	84.5

Supplementary Figure S2		MDA-MB231	MDA-MB435	BT 20	BT549	Sum - 159	Sum-1315	Average
	DMSO	100	100	100	100	100	100	100.0
	E	96.8	90.3	88.4	92.3	94.7	85.2	91.3
	N	92.1	86.9	77.9	84.7	90.2	92.3	87.4
	P400	63.8	69.2	52.6	60.4	78.6	61.9	64.4
Observed	ENP	15.6	11.5	10.8	18.2	22.7	9.5	14.7
Expected additive	ENP	56.9	54.3	36.2	47.2	67.1	48.7	51.7

Supplementary Figure S3A		MDA-MB231	MDA-MB435	BT 20	BT549	Sum - 159	Average	SF 3B	MDA-MB231	MDA-MB435	BT 20	BT549	Sum - 159	Average
	DMSO	100	100	100	100	100	100.0	DMSO	100	100	100	100	100	100.0
	V	85	82	78	84	94	84.6	B	92	91	86	89	94	90.4
	N	90	89	76	88	95	87.6	N	91	90	78	84	91	86.8
	P	95	96	90	97	93	94.2	P	90	94	85	94	89	90.4
	VN	80	75	70	83	84	78.4	BN	82	88	73	76	81	80.0
	VP	70	72	68	71	78	71.8	BP	90	85	83	90	92	88.0
	NP	84	80	82	90.4	84	84.1	NP	86	81	84	86	92	85.8
Observed	VNP	21.6	24	17	19	21	20.5	BNP	26	33.7	35	34	32	32.1
Expected additive	VNP	72.7	70.1	53.4	71.7	83.0	70.2	BPN	75.3	77.0	57.0	70.3	76.1	71.2

Supplementary Figure S3C		MDA-MB231	MDA-MB435	BT 20	BT549	Sum 159	Average
	DMSO	100	100	100	100	100	100.0
	E	92	89	87	100	91	91.8
	N	91	90	78	85	90	86.8
	P	93	91.5	83	92	90	89.9
	EN	93	89	75	78	88	84.6
	EP	90	85	83	90	92	88.0
	NP	91	86	80	82	89	85.6
Observed	ENP	16	20.7	15.5	24	34.6	22.2
Expected additive	ENP	77.9	73.3	56.3	78.2	73.7	71.9

Supplementary Table S1. Survival of TNBC cells with HDAC-I, HSP90-I and niclosamide.

Supplementary Figure S4A	IBC02	IBC03	KPL4	SUM149	SUM190
DMSO	101	100	100	103	100
Entinostat 100	21	61	45	99	159
Entinonostat150	12	35	20	102	223
Niclosamide100	20	46	91	113	135
Pimitespib100	107	89	89	115	114
Pimitespib200	114	81	118	172	102
E100N100P100	**0**	32	32	86	208
Expected Additive	4	25	36	128	244
E100N100P200	**0**	12	**15**	103	139
Expected Additive	3	14	48	192	218
E150N100P100	**0**	**13**	**7**	78	262
Expected Additive	5	23	16	132	342
E150N100P200	**0**	**9**	**5**	128	177
Expected Additive	3	13	22	197	306
**Supplementary Figure S4B**	**IBC02**	**IBC03**	**KPL4**	**SUM149**	**SUM190**
DMSO	100	99	100	105	100
Belinostat500	14	91	91	71	95
Belinostat1000	0	45	23	21	107
Niclosamide100	17	35	104	74	133
Pimitespib100	114	89	102	111	112
Pimitespib200	105	81	136	158	100
B500N100P100	**0**	40	58	37	91
Expected Additive	3	28	97	59	142
B1000N100P100	**0**	**12**	42	37	62
Expected Additive	0	14	25	17	160
B500N100P200	**0**	**7**	**17**	**6**	79
Expected Additive	3	26	130	83	126
B1000N100P200	0	**7**	**15**	**8**	60
Expected Additive	0	13	33	24	143

Supplementary Table S2. Survival of inflammatory breast cancer cells with HDAC-I, HSP90-I and niclosamide.

Rank	NCT Number	Drug 1	Drug 2	Drug 2 target	Phase	Status
1	NCT00413075	Belinostat			Phase 1	Completed
2	NCT00413322	Belinostat	5-FU	DNA synthesis	Phase 1	Completed
3	NCT04315233	Belinostat	Ribociclib	Cyclin D1/CDK4 /6	Phase 1	Recruiting
4	NCT04703920	Belinostat	Talazoparib	PARP	Phase 1	Recruiting
5	NCT03432741	Belinostat			Phase 1	Recruiting
7	NCT00627627	Belinostat			Phase 1|2	Withdrawn
6	NCT00817362	Belinostat	Trastuzumab	HER2	Phase 2	Terminated
8	NCT02453620	Entinostat	Nivolumab	PD-L1/2	Phase 1	Active, not recruiting
13	NCT02833155	Entinostat			Phase 1	Completed
14	NCT02820961	Entinostat	Exemestane	ER	Phase 1	Completed
15	NCT01594398	Entinostat			Phase 1	Completed
16	NCT01434303	Entinostat	Lapatinib	HER2	Phase 1	Completed
17	NCT02623751	Entinostat			Phase 1	Completed
18	NCT02897778	Entinostat			Phase 1	Completed
19	NCT00020579	Entinostat			Phase 1	Completed
24	NCT03473639	Entinostat			Phase 1	Recruiting
26	NCT04296942	Entinostat	Adotrastuzumab	HER2	Phase 1	Terminated
27	NCT00754312	Entinostat			Phase 1	Terminated
20	NCT02708680	Entinostat	Atezolizumab	PD-L1	Phase 1| 2	Completed
9	NCT03280563	Entinostat			Phase 1|2	Active, not recruiting
10	NCT01349959	Entinostat	Azacitidine	DNA methyltransferase	Phase 2	Active, not recruiting
21	NCT00828854	Entinostat			Phase 2	Completed
22	NCT00676663	Entinostat	Exemestane	ER	Phase 2	Completed
23	NCT03291886	Entinostat			Phase 2	Completed
25	NCT03361800	Entinostat			Phase 2	Terminated
28	NCT01234532	Entinostat	Anastrozole	ER	Phase 2	Terminated
29	NCT02115594	Entinostat			Phase 2	Withdrawn
11	NCT03538171	Entinostat			Phase 3	Active, not recruiting
12	NCT02115282	Entinostat			Phase 3	Active, not recruiting
30	NCT00004065	Tanespimycin			Phase 1	Completed
31	NCT00773344	Tanespimycin	Trastuzumab	HER2	Phase 1|2	Completed
32	NCT00096109	Tanespimycin			Phase 2	Terminated
34	NCT01720602	Vorinostat			N/A	Completed
35	NCT01153672	Vorinostat			N/A	Completed
55	NCT01655004	Vorinostat			N/A	Unknown status
57	NCT01695057	Vorinostat			N/A	Withdrawn
36	NCT00719875	Vorinostat	Capecitabine	DNA synthesis	Phase 1	Completed
37	NCT01084057	Vorinostat	Ixabepilone	Microtubules	Phase 1	Completed
38	NCT00788112	Vorinostat			Phase 1	Completed
39	NCT00838929	Vorinostat	Radiation		Phase 1	Completed
40	NCT00045006	Vorinostat			Phase 1	Completed
46	NCT03742245	Vorinostat	Olaparib	PARP	Phase 1	Recruiting
47	NCT03878524	Vorinostat			Phase 1	Recruiting
49	NCT01249443	Vorinostat	Carboplatin		Phase 1	Terminated
41	NCT00574587	Vorinostat	Chemotherapy		Phase 1|2	Completed
42	NCT00368875	Vorinostat	Paclitaxel	Microtubules	Phase 1|2	Completed
43	NCT00258349	Vorinostat	Trastuzumab	HER2	Phase 1|2	Completed
50	NCT01118975	Vorinostat	Lapatinib	HER2	Phase 1|2	Terminated
56	NCT00416130	Vorinostat			Phase 1|2	Unknown status
33	NCT00616967	Vorinostat	Carboplatin		Phase 2	Active, not recruiting
44	NCT00365599	Vorinostat	Tamoxifen	ER	Phase 2	Completed
45	NCT00262834	Vorinostat			Phase 2	Completed
48	NCT04190056	Vorinostat	Pembrolizumab	PD-L1	Phase 2	Recruiting
51	NCT01194427	Vorinostat	Tamoxifen	ER	Phase 2	Terminated
52	NCT00132002	Vorinostat			Phase 2	Terminated
53	NCT02395627	Vorinostat			Phase 2	Terminated
54	NCT00126451	Vorinostat			Phase 2	Terminated

Supplementary Table S3. Breast cancer clinical trials with HDAC and HSP90 inhibitors.

Frequency of genetic alterations in the cell-of-origin signature genes			
	Gene Symbol	Number of Samples Altered	Percent Samples Altered
1	CUX1	14	0.4%
2	**HDAC1**	10	0.3%
3	CROCC	10	0.3%
4	EPHB3	10	0.3%
5	PCDH1	10	0.3%
6	FCHO1	9	0.3%
7	TLE6	9	0.3%
8	PLA2G6	7	0.2%
9	LMTK3	5	0.2%
10	MYO1B	5	0.2%
11	ADGRG1	4	0.1%
12	LIMK2	4	0.1%
13	SCNN1A	4	0.1%
14	SPHK2	4	0.1%
15	DKK3	3	0.1%
16	FIBCD1	3	0.1%
17	GDPD5	3	0.1%
18	KRT7	3	0.1%
19	SHKBP1	3	0.1%
20	SLC29A3	3	0.1%
21	**ZNF92**	2	0.1%
22	ABTB1	2	0.1%
23	PDLIM1	2	0.1%
24	RABGGTA	2	0.1%
25	TMEM53	2	0.1%
26	TMEM91	2	0.1%
27	**HDAC7**	1	0.0%
28	AZIN2	1	0.0%
29	ECH1	1	0.0%
30	LOXL1	1	0.0%
31	BCAS4	0	0.0%
32	COQ8B	0	0.0%

Supplementary Table S4. The frequency of genomic alterations in the cell-of-origin signature.

3,593 breast cancer samples from TCGA and Metabric are examined for mutations, structural variants and copy number changes in HDAC1, HDAC7, ZNF92 and the 29 genes with the ZNF92 binding sites (excluding alterations of unknown significance).

1) Developing New Analysis Functions for a Translational Research Platform: Extending the cBioPortal for Cancer Genomics. Unberath, P., C. Knell, H.U. Prokosch, and J. Christoph, Stud Health Technol Inform. 258: p. 46-50, 2019.
2) Integrative analysis of complex cancer genomics and clinical profiles using the cBioPortal. Gao, J., B.A. Aksoy, U. Dogrusoz, G. Dresdner, B. Gross, S.O. Sumer, Y. Sun, A. Jacobsen, R. Sinha, E. Larsson, E. Cerami, C. Sander, and N. Schultz, Sci Signal. 6(269): p. pl1, 2013.
